# Incremental changes in QRS duration as predictor for cardiovascular disease: a 21-year follow-up of a randomly selected general population

**DOI:** 10.1038/s41598-021-93024-y

**Published:** 2021-07-01

**Authors:** Xiaojing Chen, Per-Olof Hansson, Erik Thunström, Zacharias Mandalenakis, Kenneth Caidahl, Michael Fu

**Affiliations:** 1 Department of Cardiology, West China Hospital, Sichuan University, Chengdu, Sichuan China; 2 Department of Molecular and Clinical Medicine, Institute of Medicine, Sahlgrenska Academy, University of Gothenburg, Gothenburg, Sweden; 3 Department of Medicine, Geriatrics and Emergency Medicine, Sahlgrenska University Hospital, Gothenburg, Region Västra Götaland Sweden; 4 Department of Clinical Physiology, Sahlgrenska University Hospital, Gothenburg, Region Västra Götaland Sweden; 5 Department of Molecular Medicine and Surgery, Karolinska Institutet, Karolinska Universtity Hospital, Stockholm, Sweden

**Keywords:** Public health, Cardiology, Risk factors

## Abstract

The QRS complex has been shown to be a prognostic marker in coronary artery disease. However, the changes in QRS duration over time, and its predictive value for cardiovascular disease in the general population is poorly studied. So we aimed to explore if increased QRS duration from the age of 50–60 is associated with increased risk of major cardiovascular events during a further follow-up to age 71. A random population sample of 798 men born in 1943 were examined in 1993 at 50 years of age, and re-examined in 2003 at age 60 and 2014 at age 71. Participants who developed cardiovascular disease before the re-examination in 2003 (n = 86) or missing value of QRS duration in 2003 (n = 127) were excluded. ΔQRS was defined as increase in QRS duration from age 50 to 60. Participants were divided into three groups: group 1: ΔQRS < 4 ms, group 2: 4 ms ≤ ΔQRS < 8 ms, group 3: ΔQRS ≥ 8 ms. Endpoints were major cardiovascular events. And we found compared with men in group 1 (ΔQRS < 4 ms), men with ΔQRS ≥ 8 ms had a 56% increased risk of MACE during follow-up to 71 years of age after adjusted for BMI, systolic blood pressure, smoking, hyperlipidemia, diabetes and heart rate in a multivariable Cox regression analysis (HR 1.56, 95% CI:1.07–2.27, P = 0.022). In conclusion, in this longitudinal follow-up over a decade QRS duration increased in almost two out of three men between age 50 and 60 and the increased QRS duration in middle age is an independent predictor of major cardiovascular events.

## Introduction

The QRS complex on the standard, resting 12-lead electrocardiogram (ECG) is an imaging of the electrical impulse propagation through the conduction system and the ventricular myocardium. Previous studies have demonstrated that a normal 12-lead ECG is a relatively sensitive and specific marker of normal conduction and left ventricular function. QRS duration on 12-lead ECG at rest is related to all-cause mortality, cardiovascular disease (CVD) mortality and heart failure in the general population^1–4^ as well as in patients with myocardial infarction or heart failure^5–7^. Prolonged QRS duration (> 120 ms) has also been linked to left ventricular systolic dysfunction^8^, increased LV end-systolic volume^9^, and LV hypertrophy^9,10^, each of which are associated with CVD. However, the dynamics in QRS duration over time and its impact on long-term cardiovascular events in the general population remains to be studied. Since QRS duration on 12-lead ECG is an easily available clinical measurement, knowledge of the importance of changes in QRS duration might be helpful to identify high-risk subjects. We hypothesized that increasing QRS duration would be independently associated with incident CVD in a general population sample.

## Methods

### Study population

“The study of Men Born in 1943” is a longitudinal, prospective, population-based study of men born in 1943 and living in the city of Gothenburg in western Sweden^11^. In 1993, the local tax authority generated a random sample of 50% of all men who were born in 1943 and were resident in the city of Gothenburg. These1463 men were invited to participate in an examination, and 798 men (54.5%) agreed to participate. Informed consent was obtained from all participants. The study complied with the Declaration of Helsinki, and the study protocol was approved by the Ethics Committee of Gothenburg (DNR 157–93, 0067–03 and 649–13).

### Data collection

All 798 men who were examined in 1993 were invited to a re-examination in 2003 all at age 60 years, where 655 out of 773 who were still alive (84.7%) were re-examined, and in 2014, at the age of 71 years 536 out of 688 men still alive (77.9%) were re-examined. The follow-up procedure is previously described in detail^12–14^.

At each examination, clinical examination and laboratory analysis were performed. Data on smoking habits, leisure time physical activity, previous disease and pharmacological treatment were collected by questionnaire. Leisure time physical activity was assessed by the Saltin-Grimby questionnaire^15^, and coded as: 1 = sedentary (physically inactive), 2 = some light physical activity such as walking, riding a bicycle, or light gardening for at least 4 h per week, 3 = regular, moderate physical activity for a minimum of 3 h per week; and 4 = regular, vigorous physical training. Men who were current smoker or had quit smoking < 1 month before the examination were categorized as smokers. Former smokers were defined as those who had quit smoking ≧1 month previously and never-smoker as those who had never used cigarettes, cigars, or a pipe on the regular basis.

### Clinical examination

Height and weight (in light indoor clothing) were measured and body mass index (weight in kg/ height in m^2^) was calculated. A standard cuff and a mercury manometer were used to measure blood pressure. Hypertension was diagnosed based on either medical history with current antihypertensive therapy or current blood pressure ≧140 (systolic), ≧90 mmHg (diastolic), or both. Fasting venous blood samples were drawn in the morning for analysis of blood glucose, serum cholesterol, and triglyceride measurements were analysed at the local accredited laboratory. Hyperlipidemia was defined as total cholesterol > 6.2 mmol/L or using lipid-lowering medication. Diabetes mellitus was defined as fasting blood glucose > 7 mmol/L or using oral medication and/or insulin.

### ECG recordings and measurement of QRS duration

Heart rate was measured by 12-lead ECG in the supine position in 1993, 2003 and 2014. Paper speed was 50 mm/s and the calibration was 1 mV:10 mm. All ECGs were evaluated by a physician, who was blinded to all clinical data. The QRS- duration was automatically measured by the ECG.

Participants who had developed CVD before 2003 were excluded from the study, however, we did not exclude individuals with bundle branch blocks. The change of QRS (ΔQRS) was defined as QRS duration in 2003 minus QRS duration in 1993. ΔQRS < 0 ms, 0– < 4 ms, 4–8 ms and > 8 ms represented the approximate quartiles for QRS change in our database. Since long ΔQRS was considered as most clinically important, the lower two ΔQRS quartiles were combined to one group: thus group 1 had ΔQRS < 4 ms, group 2 had 4 ms ≤ ΔQRS < 8 ms, and group 3: ΔQRS ≥ 8 ms.

### Follow-up procedures and end-point

All men in the study were followed from the baseline examination, 1993, until August 31, 2014. Data on hospital admissions were obtained for the complete study population from the National Hospital Discharge Register, covering all hospital admissions in the country, for all participants from 1993 to 2014 and by collecting and reviewing medical charts. Mortality data were obtained from the Cause of Death Register.

As endpoint we used any major adverse cardiovascular event (MACE) defined as the occurrence of myocardial infarction, heart failure, death resulting from coronary heart disease, stroke, intermittent claudication, revascularization procedure, death resulting from coronary heart disease, or other cardiovascular death.

### Statistical analysis

For the baseline characteristics of participants, continuous variables were described by mean and standard deviation (SD), while categorical variables were reported as frequencies (%). Differences in the distribution of baseline characteristics in different QRS categories were examined using Kruskal–Wallis test (continuous variables) and chi-square tests (categorical variables).

Crude incidence rates were expressed as event rates, calculated as the number of events divided by the sum of follow-up years per 1000 person-years. Univariable and multivariable-adjusted Cox proportional hazard models were used to examine the associations of different QRS duration change with outcomes, using group 1 (ΔQRS < 4 ms) as reference group, unadjusted and multivariate-adjusted hazard ratios (HR) and 95% confidence intervals (95%CI) were estimated for each QRS group. The multivariable model was adjusted for body mass index, systolic blood pressure, smoking, hyperlipidemia, diabetes and heart rate.

All statistical tests were 2-tailed with 95% confidence levels and p values of < 0.05 were considered significant. IBM SPSS Statistics for Windows, Version 22 was used for data analyses.

## Results

**Figure 1:**
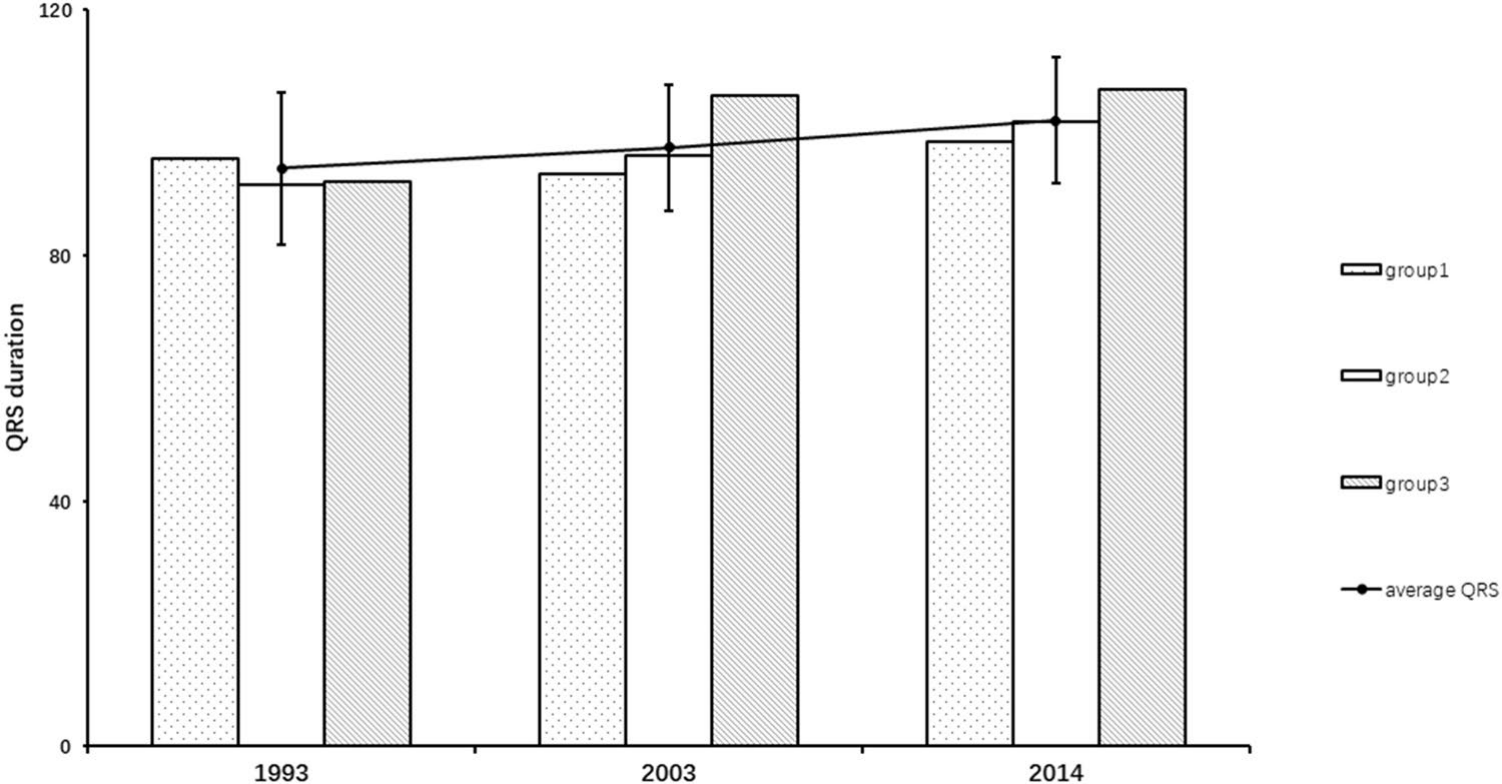
Baseline, final and ΔQRS width in participants reporting in different QRS change groups (group 1: ΔQRS < 4 ms, group 2: 4 ms ≤ ΔQRS < 8 ms, group 3: ΔQRS ≥ 8 ms).

Of the 798 men, 86 had developed cardiovascular disease before the re-examination in 2003 while another 127 did not participate in the re-examination and consequently had missing value of QRS duration in 2003. These 213 men were excluded, leaving 585 men in the final study. Of those, 379 (64.7%) men had an increase in QRS duration. Changes in QRS duration over the 21-year follow-up are shown in Fig. 1. The mean QRS duration in 1993, 2003 and 2014 were 93.8 ± 12.1 ms, 97.2 ± 12.3 ms and 101.6 ± 18.3 ms, respectively. Mean QRS duration increased from 93.8 ms at baseline to 101.6 ms at the examination 21 years later (Fig. 1).

### Baseline characteristics

Baseline characteristics were similar among the three groups, and participants in group 3 only differed by having slightly higher triglycerides compared to men in group 1 and 2 (Table 1).

**Table 1:** Baseline characteristics (1993) according to different QRS change group.

	QRS change in 2003 and 1993			P value
	Group 1 ΔQRS < 4 ms (n = 288)	Group 2 4 ms ≤ ΔQRS < 8 ms (n = 150)	Group 3 ΔQRS ≥ 8 ms (n = 147)	
QRS width in1993, ms	95.7 ± 12.2	91.4 ± 7.9	91.9 ± 12.2	< 0.001
QRS width in 2003, ms	93.3 ± 10.1	96.2 ± 7.9	105.8 ± 15.4	< 0.001
QRS width in 2014, ms	98.5 ± 15.7	101.8 ± 16.9	106.7 ± 20.7	< 0.001
**Clinical characteristic**				
Smoking (%)				
Never smoking	108 (37.5)	46 (30.7)	51 (34.7)	0.487
Previous smoking	110 (38.1)	62(41.3)	52 (35.4)	
Current smoking	70 (24.2)	42(28.0)	44 (29.9)	
Sedentary lifestyle	35 (12.1)	24(16.0)	16 (10.9)	0.371
Mental stress	37 (12.8)	19(12.8)	22 (14.9)	0.803
BMI, kg/m^2^	25.8 ± 3.3	26.2 ± 3.5	26.3 ± 3.0	0.186
Waist circumference, cm	93.7 ± 8.7	95.4 ± 9.5	94.8 ± 8.4	0.150
Systolic BP, mmHg	128.2 ± 17.1	127.4 ± 16.8	127.7 ± 15.7	0.870
Diastolic BP, mmHg	83.6 ± 10.5	83.1 ± 9.6	84.0 ± 10.1	0.729
Heart rate, bpm	65.3 ± 11.8	65.4 ± 11.5	66.5 ± 11.9	0.569
**Medical history (%)**				
Hypertension	26 (9.0)	17 (11.4)	16 (11.0)	0.675
Hyperlipidemia	21 (7.3)	15 (10.1)	15 (10.3)	0.461
Diabetes	7 (2.4)	3 (2.0)	2 (1.4)	0.760
**Laboratory characteristic**				
Cholesterol, mmol/L	5.8 ± 1.0	5.9 ± 0.9	5.9 ± 1.0	0.300
Triglycerides, mmol/L	1.5 ± 0.8	1.6 ± 0.9	1.8 ± 1.5	0.024
eGFR	100.0 ± 16.1	103.3 ± 18.6	102.8 ± 17.7	0.097

### The relationship between QRS duration change and outcome

**Figure 2:**
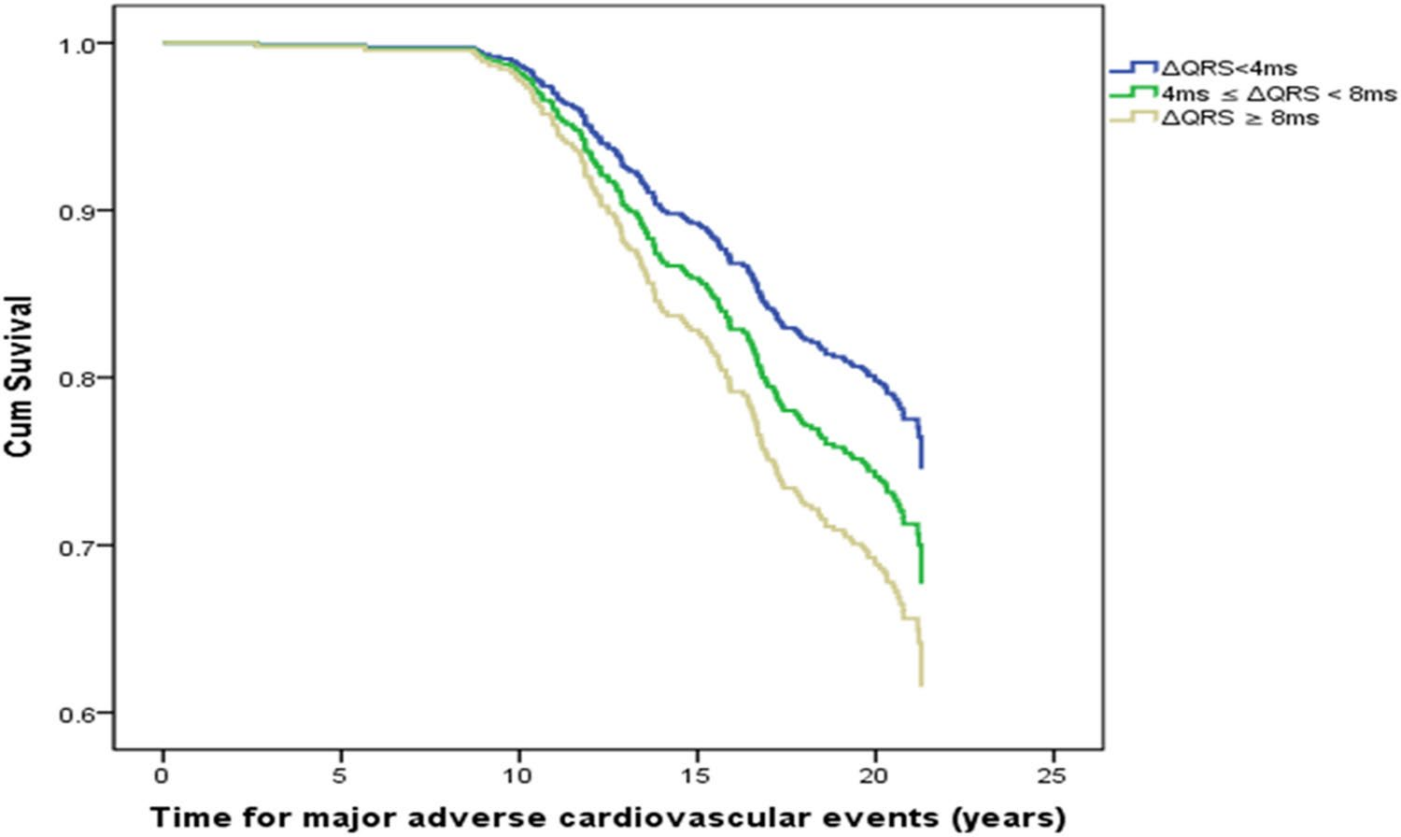
Adjusted risk for MACE according to different QRS duration change group.

The QRS change observed between 1993 and 2003 was not related to the 2014 data of systolic, diastolic or mean blood pressure levels (data not shown), but to increasing echocardiographically calculated LV mass (91.1 ± 22.4, 94 ± 23.9 and 99.3 ± 24.5 g, in the three ΔQRS groups, p = 0.01). Of the 585 participants included in the analysis, 155 (26.4%) experienced MACE, during a follow-up of 11 years from 60 to 71 years of age. The occurrence of MACE was not related to the absolute QRS duration in 2003. Figure 2 shows time to MACE for the three groups of change in QRS duration. In univariate analysis, men in group 2 (QRS change 4 ms ≤ ΔQRS < 8 ms) had an HR of 1.27 (95% CI: 0.86–1.88) and men in group 3 (increase in QRS duration ≥ 8 ms ) an HR of 1.59 (95% CI: (1.10–2.31) for MACE compared to group 1 (with ΔQRS < 4 ms), Table 2. The HRs were not much changed by adjustments in multivariate Cox proportional hazard model including 2003 data on BMI, systolic blood pressure, smoking, hyperlipidemia, diabetes and heart rate, yielding for group 2 an HR of 1.26 (95% CI: 0.85–1.86) and group 3 an HR of 1.56 (95% CI 1.07–2.27), Table 2.

**Table 2:** Outcome data according to different ΔQRS group.

Outcome	Model	QRS change from 1993 to 2003				
		Group1 (n = 288) ΔQRS < 4 ms	Group 2 (n = 150) 4 ms ≤ ΔQRS < 8 ms		Group 3(n = 147) ΔQRS ≥ 8 ms	
		Ref	HR (95% CI)	P value	HR (95% CI)	P value
MACE	No events (%)	65 (22.6)	41 (27.3)		49 (33.3)	
	Unadjusted	1.00 (*ref*)	1.27 (0.86–1.88)	0.232	1.59 (1.10–2.31)	0.014
	Adjusted	1.00 (*ref*)	1.26 (0.85–1.86)	0.249	1.56 (1.07–2.27)	0.021

Adjusted for: body mass index, systolic blood pressure, hyperlipidemia, diabetes and heart rate.

## Discussion

This longitudinal follow-up of a general population sample showed a prolongation of the QRS duration in most men without cardiovascular disease at baseline between the age 50 and 71. Individuals with increased ΔQRS between1993 and 2003 were at increased risk for developing cardiovascular disease in the next eleven years the risk of MACE were highest for participants with the highest increase in ΔQRS.

The QRS duration represents the time taken for the electrical wave of myocardial depolarization to move from the His-Purkinje system through the ventricular myocardium and physiological or pathological changes in the myocardial conduction velocity could explain some of the variation in QRS duration. The QRS duration has been found to be dependent on both LV mass and LV volume in a large group of subjects at high CV risk^9^. In our study, we found a relation between increasing QRS duration during a 10-year period, and the LV mass another 11 years later.

It is well known that among patients with established heart disease, a prolonged QRS duration on ECG is an independent predictor of adverse outcome^4,5^. In most patients with systolic left ventricular dysfunction, increased QRS duration is associated with a worse prognosis^16,17^. Among patients with coronary artery disease, QRS prolongation has been associated with a 50% increased risk of both arrhythmic and total mortality^18^. In a study of 1227 patients with suspected coronary artery disease referred for noninvasive evaluation of myocardial ischemia, QRS duration was found to be an independent predictor of cardiac death and nonfatal infarction^19^.

When referred to the general population, prolonged QRS duration usually indicates myocardial changes in response to underlying CVD and has been associated with decreased LV systolic and diastolic function, arrhythmia, ischemic cardiac disease, and LV hypertrophy^9^. However, few studies have examined change in QRS duration and its prognostic value in a general population sample, and results from previous studies are deverging. In a large Veteran's Administration study of more than 45 000 patients with an average age of 56 ± 15 years (90% were male), investigators found that every 10-ms prolongation of the QRS duration was associated with an 18% increase in risk for cardiovascular death^1^. The LIFE study reported that QRS duration predicts mortality and, importantly, sudden cardiac death in hypertensive patients on intensive medical therapy^20^. The Strong Heart Study found that QRS duration was independently associated with incident MACE, CAD, and myocardial infarction in American Indian women, but not in men^21^. Conversely, one prior study , on participants in the Framingham Heart Study with ages ranging from 44 to 78 years, reported no association between age-adjusted incidence of myocardial infarction, angina pectoris, and coronary death and baseline QRS prolongation in men and women^22^.The DIAMOND-HF study investigated the importance of incremental change in QRS duration over time on several echocardiographic outcomes and found that increment in QRS duration over time was significantly associated with deterioration of ventricular function and increase in filling pressures, and that QRS duration over time was superior to baseline QRS only in predicting a worse outcome^8^. Change in QRS duration over time was strongly associated with mortality^23^. In our study, we found that individuals with increased ΔQRS over ten years, from age 50 to 60, were at increased risk for developing cardiovascular disease during the next eleven years, but the absolute QRS duration in 2003 had no relationship with the CV outcome within another 11 years (until 2014). Thus, the present study suggests that monitoring the change in QRS duration over time can add independent prognostic information.

## Strength and limitation

Strengths of the present study include the prospective design, the random sample of men from the general population of uniform age, and the long follow-up time of 21 years. However, there are also limitations: first, we only included men of same age and similar ethnicity, and the conclusion cannot be generalized to other populations; second, we did not exclude individuals with bundle branch blocks as our intention of this paper was not QRS morphology and duration at baseline, but to evaluate importance of incremental changes of QRS duration during a 10-year period (1993–2003). Moreover, QRS prolongation occurs in many settings including bundle branch block, non-specific intraventricular conduction delay, preexcitation, myocardial hypertrophy or storage diseases etc., with divergent mechanisms. Avoiding exclusions should make it easier to generalize our findings.

## Conclusion

In conclusion, our study shows that in a general male population without known cardiovascular disease at baseline, increased ΔQRS during a 10-year period is a predictor of MACE during a successive 11-year follow-up. Our findings indicate the potential prognostic benefit of monitoring the ΔQRS over time as a means of selecting individuals who might benefit from preventive measures.
